# Localization of the Epileptogenic Foci in Tuberous Sclerosis Complex: A Pediatric Case Report

**DOI:** 10.3389/fnhum.2014.00175

**Published:** 2014-03-26

**Authors:** Alexander Hunold, Jens Haueisen, Banu Ahtam, Chiran Doshi, Chellamani Harini, Susana Camposano, Simon K. Warfield, Patricia Ellen Grant, Yoshio Okada, Christos Papadelis

**Affiliations:** ^1^Institute of Biomedical Engineering and Informatics, Ilmenau University of Technology, Ilmenau, Germany; ^2^Fetal-Neonatal Neuroimaging and Developmental Science Center, Boston Children’s Hospital, Harvard Medical School, Boston, MA, USA; ^3^Department of Newborn Medicine, Boston Children’s Hospital, Harvard Medical School, Boston, MA, USA; ^4^Department of Neurology, Boston Children’s Hospital, Harvard Medical School, Boston, MA, USA; ^5^Department of Radiology, Boston Children’s Hospital, Harvard Medical School, Boston, MA, USA; ^6^Computational Radiology Laboratory, Boston Children’s Hospital, Harvard Medical School, Boston, MA, USA

**Keywords:** electroencephalography, epileptogenic zone, equivalent current dipole, magnetoencephalography, pediatric epilepsy, tuberous sclerosis complex

## Abstract

Tuberous sclerosis complex (TSC) is a rare disorder of tissue growth and differentiation, characterized by benign hamartomas in the brain and other organs. Up to 90% of TSC patients develop epilepsy and 50% become medically intractable requiring resective surgery. The surgical outcome of TSC patients depends on the accurate identification of the epileptogenic zone consisting of tubers and the surrounding epileptogenic tissue. There is conflicting evidence whether the epileptogenic zone is in the tuber itself or in abnormally developed surrounding cortex. Here, we report the localization of the epileptiform activity among the many cortical tubers in a 4-year-old patient with TSC-related refractory epilepsy undergoing magnetoencephalography (MEG), electroencephalography (EEG), and diffusion tensor imaging (DTI). For MEG, we used a prototype system that offers higher spatial resolution and sensitivity compared to the conventional adult systems. The generators of interictal activity were localized using both EEG and MEG with equivalent current dipole (ECD) and minimum norm estimation (MNE) methods according to the current clinical standards. For DTI, we calculated four diffusion scalar parameters for the fibers passing through four ROIs defined: (i) at a large cortical tuber identified at the right quadrant, (ii) at the normal appearing tissue contralateral to the tuber, (iii) at the cluster formed by ECDs fitted at the peak of interictal spikes, and (iv) at the normal appearing tissue contralateral to the cluster. ECDs were consistently clustered at the vicinity of the large calcified cortical tuber. MNE and ECDs indicated epileptiform activity in the same areas. DTI analysis showed differences between the scalar values of the tracks passing through the tuber and the ECD cluster. In this illustrative case, we provide evidence from different neuroimaging modalities, which support the view that epileptiform activity may derive from abnormally developed tissue surrounding the tuber rather than the tuber itself.

## Introduction

Tuberous sclerosis complex (TSC) is a multi-system, autosomal dominant disorder (Crino et al., 2006) with a prevalence of 7–12/100,000 (O’Callaghan et al., 1998). TSC children present severe neurological symptoms that are mainly related to the cortical tubers, which occur in 80% of these patients (Crino et al., 2006). Approximately 90% of TSC children develop epilepsy; nearly two-thirds of patients have seizure onset within the first year of their life (Curatolo et al., 2002). In these patients, infantile spasms are the most common type of seizures (Chiron et al., 1997) with an onset of as early as 4 months of age. Partial seizures are often seen, whereas generalized seizures are relatively rare (Chiron et al., 1997; Kotagal, 2001).

Approximately 50% of the TSC-related epilepsy cases are refractory to pharmacological therapy (Jansen et al., 2006). Resective epileptic surgery is thus considered for controlling the seizures. Although surgical outcome differs in these patients, many studies report a positive surgical outcome in patients who have undergone surgical resection of the tubers (Bebin et al., 1993; Avellino et al., 1997; Karenfort et al., 2002; Romanelli et al., 2004). A recent systematic review reported that with resective surgery 57% of children achieve seizure freedom and another 18% experience a reduction (>90%) in seizure frequency at 1 year follow-up (Jansen et al., 2007).

The success of resective surgery in TSC patients depends on the accurate identification of the entire epileptogenic tissue (Tran et al., 1997). Multiple potentially epileptogenic tubers are usually present in TSC children and the task is to differentiate those associated with epileptiform activity from the “silent” ones. Another task is to find out whether epileptogenicity derives from the tubers themselves or the abnormally developed surrounding cortex. Epileptogenic tubers may also occur in eloquent cortex, potentially rendering surgical treatment more difficult. There is conflicting evidence so far regarding these issues. Surgical studies report that the resection of large, calcified tubers is associated with a marked improvement in the seizure profiles of TSC patients (Guerreiro et al., 1998; Koh et al., 2000; Lachhwani et al., 2005). A surgical series using intraoperative electrocorticography (ECoG) indicated that the epileptiform discharges were localized within the cortical tubers (Guerreiro et al., 1998). Other ECoG studies found that the electrographic tubers were silent, and it was the surrounding neural tissue that was epileptogenic (Major et al., 2009).

Currently, the epileptogenic zone is conventionally identified using a combination of invasive and non-invasive imaging modalities. Invasive intracranial recordings serve as gold standard for the localization of the epileptogenic zone; however they are costly, can be difficult due to the cooperation of the child, carry some risk for infection and bleeding (Onal et al., 2003), and neurological damage (Zaccariotti et al., 1999). Intracranial recordings explore limited areas and hence, the success of such studies depends on the hypothesis formed by the results of the non-invasive tests. Scalp ictal EEG can be non-localizing in a significant proportion of children.

Advanced neuroimaging techniques can play a significant role in defining the epileptogenic zone in patients with TSC and multiple lesions (Jacobs et al., 2008; Sugiyama et al., 2009). Usually, data from several imaging modalities, such as electroencephalography (EEG), magnetoencephalography (MEG), diffusion tensor imaging (DTI), single-photon emission computed tomography (SPECT), and positron emission tomography (PET) must be integrated for an accurate presurgical localization. MEG (Kamimura et al., 2006; Xiao et al., 2006; Widjaja et al., 2010) or EEG data (Leal et al., 2008; Ochi et al., 2011; Kargiotis et al., 2013) are frequently reported in the literature. Very few reports present simultaneous MEG and EEG recordings (Iida et al., 2005; Sugiyama et al., 2009) in TSC-related pediatric epilepsy patients. Few other studies report mixed pediatric and adult patient cohorts (Jansen et al., 2006; Wu et al., 2006; Canuet et al., 2008). For source analysis, previous investigations of epileptic foci in TSC patients used various methods for the identification of the epileptic active areas, such as synthetic aperture magnetometry (SAM) (Xiao et al., 2006; Sugiyama et al., 2009), equivalent current dipoles (ECDs) (Iida et al., 2005; Kamimura et al., 2006; Wu et al., 2006; Xiao et al., 2006; Leal et al., 2008; Sugiyama et al., 2009; Widjaja et al., 2010), or multiple signal classification (MUSIC) (Jansen et al., 2006).

Magnetoencephalography is considered as one of the most promising techniques that can help in the non-invasive localization of epileptiform activity in the TSC-related epilepsy population. It provides an excellent localization accuracy of few millimeters for superficial sources (Leahy et al., 1998; Papadelis et al., 2009). Two recent studies presented evidence that MEG is superior compared to other neuroimaging techniques in the identification of the epileptogenic tissue in TSC patients. Wu et al. (2006) showed that MEG was better, in terms of sensitivity, specificity, and accuracy, compared to the ictal video-EEG in the identification of the epileptogenic zone in TSC patients. Jansen et al. (2006) found that epileptiform activity in patients with TSC and epilepsy detected with MEG was closer to a presumed epileptogenic tuber than the epileptiform activity detected with EEG.

DTI is used to detect microstructural changes in cortical development malformations (Widjaja et al., 2010) and to evaluate cortical tubers and normal appearing white matter (NAWM) in TSC patients (Peng et al., 2004; Makki et al., 2007). Previous studies reported increased apparent diffusion coefficient (ADC) and decreased fractional anisotropy (FA) values in the white matter lesions and the perilesional white matter compared to the contralateral NAWM in patients with TSC (Peng et al., 2004; Karadag et al., 2005). TSC patients with epilepsy have also been reported to have white matter abnormalities suggested to be indicative of cortical dysplasia or impaired myelin development due to seizures (Dwyser and Wasterlain, 1982; Jansen et al., 2003; Song et al., 2003; Widjaja et al., 2010), although the radial direction of the white matter abnormalities is more in keeping with dysplasia. Decreased FA (Widjaja et al., 2010) and increased ADC (Jansen et al., 2003) values were found for the cortical tubers within the epileptogenic zone compared to the cortical tubers in the non-epileptogenic zone in TSC patients with epilepsy.

In this case report, we examine a 4-year-old patient with TSC-related epilepsy by using pediatric MEG, EEG, MRI, and DTI. By using MEG and EEG, we aimed to identify whether epileptiform activity in this patient was derived from the tuber itself or its surrounding cortex and quantify the geometric configuration between source localization and tuber margin. For MEG, we used the BabySQUID system that has been especially designed for pediatric use. BabySQUID offers higher spatial resolution (3×) and sensitivity (2×) compared to conventional adult MEG (Okada et al., 2006). By using DTI, we aimed to detect white matter differences between the fibers passing through the calcified tuber, the contralateral NAWM, and the irritative zone as this was indicated by MEG and EEG.

## Materials and Methods

### Clinical presentation

We studied a 4-year-old female patient with refractory epilepsy as a result of TSC. The patient had an uncomplicated perinatal history. Her first seizure (infantile spasms) occurred when she was 4 months old. Cardiac rhabdomyoma, revealed by echocardiography, resulted in the diagnosis of TSC. A *TSC1* mutation was found. MRI revealed multiple tubers in the bilateral frontal, parietal, and occipital lobes. A large calcified tuber was identified in the right parieto-occipital lobe. The following years the patient presented frequent gelastic seizures (smiling for ~5 s) with a daily frequency at maximum.

Previous routine EEG studies at the age of 3 years from this patient reported very frequent right occipital/posterior temporal sharp waves (P8 and O2). Less frequent spikes and sharp waves were identified at the left frontal (F3) as well as right fronto-temporal regions. Intermittent slowing in the left frontal and right posterior quadrant was observed. Ambulatory EEG at the age of 3 years detected five electrographic seizures arising from the right occipital region. Long-term monitoring captured six gelastic seizures with apparent left frontal onset. Very frequent interictal spikes were seen in the right posterior quadrant and left anterior quadrant occurring independently. Although the ictal EEG onset was noted in the left frontal region, source analysis of the ictal onset revealed a more complex pattern involving both left frontal and right occipito-parietal activity. More specifically, it was noted that there were frequent right posterior temporal interictal spikes that diminish just as frequent low amplitude left frontal spikes were observed, which precede clinical seizure onset. These spikes pause and then left frontal spikes reappear with slightly different spatial and topographic distribution. The source analysis concluded that although the seizures appear to arise from the left frontal source, the right-sided abnormality could serve to facilitate the left frontal seizure source. The alpha-methyl-tryptophan PET scan showed increased uptake over the right parieto-temporo-occipital cortex. It was decided that intracranial EEG was needed to cover both regions. However, patient became seizure free with the introduction of a different antiepileptic medication. Hence, at this time the patient is being followed up closely without a surgical evaluation.

### Recordings

**Figure 1:**
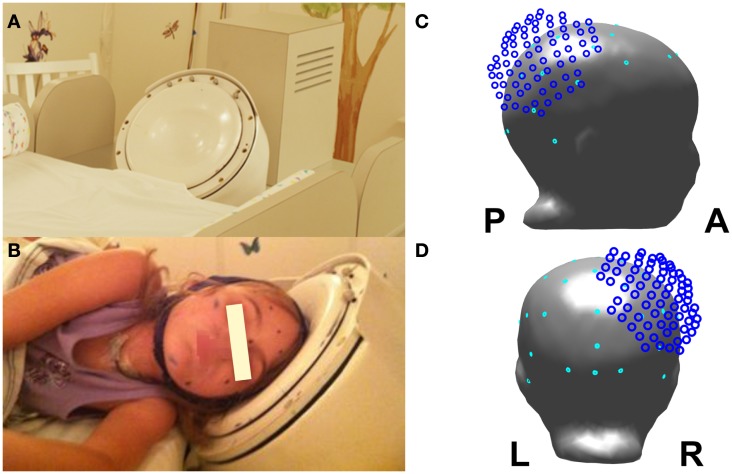
**Experimental recordings setup: (A) view of the BabySQUID headrest; (B) the epilepsy patient wearing the pediatric EEG cap and placing her head next to the BabySQUID headrest; (C) lateral view of patient’s head model with the relative location of MEG sensors as it was positioned during the recordings (MEG sensors in dark blue and EEG sensor in cyan); (D) posterior view of the head model, the MEG sensors, and EEG electrodes**.

Multichannel MEG and EEG signals were simultaneously recorded from the patient for 10 min during sleep when the patient was 4 years old. MEG recordings were performed using BabySQUID (Tristan Technologies Inc., San Diego, USA). The system is equipped with 74 asymmetric axial first-order gradiometers covering the brain partially. A detailed description of BabySQUID can be found in Okada et al. (2006). The system is accommodated in a single-layer magnetically shielded room (MSR) located at the Radiology Suite of Boston Children’s Hospital (BCH) at Waltham, MA, USA. EEG recordings were performed using a 32-channel EEG cap specially designed for pediatric use (WaveGuard cap with extended 10–20 layout; ANT b.V., Enschede, Netherlands) with common average reference. Both MEG and EEG data were sampled at 1024 samples per second. Electrocardiography (ECG) data were also recorded simultaneously at the same sampling rate. Clinical data were obtained and research MEG and EEG data were acquired and analyzed after explicit parental consent under a protocol approval by the local institutional review board. Figure 1 shows the setup of the combined MEG and EEG measurements. During the recordings, the patient’s head was placed over the sensor array, which fully covered the right parieto-occipital quadrant where the large classified tuber was located. The patient was sleeping during the entire recording and no movement was observed or recorded during the whole experiment. The co-registration was performed at the beginning and at the end of the recording session, which lasted for 10 min. The observed head movement was <2 mm. The co-registration procedure followed in our lab in epilepsy patients is described in Papadelis et al. (2013).

### MRI acquisition

The MRI data were collected at a 3T Siemens Tim Trio MR scanner at BCH when the subject was 4 years old. No sedation or medication was used during the MRI scan. The imaging protocol consisted of structural and diffusion-weighted sequences. The first structural sequence was a T1-weighted high-resolution magnetization-prepared rapid-acquisition gradient-echo (MPRAGE) acquisition, which used volumetric EPI navigators for real time motion correction [voxel size (mm) = 1 × 1 × 1; field of view (FOV) = 19.2 cm; echo time (TE) = 1.74 ms; repetition time (TR) = 2530 ms; flip angle = 7°]. The second structural sequence was a T2-weighted turbo spin-echo FLAIR sequence (TSE-FLAIR) [voxel size (mm) = 0.6250 × 0.6250 × 4.0000; FOV = 25.6 cm; TE = 1.37 ms; TR = 9000 ms; flip angle = 150°]. The diffusion sequence (prescribed axially) used echo-planar (EP) readouts [voxel size (mm) = 1.7 × 1.7 × 2.0; FOV = 22 cm; TE = 78 ms; TR = 8100 ms; flip angle = 90°; 30 gradient diffusion directions at *b* = 1000 s/mm^2^; five acquisitions with *b* = 0 s/mm^2^].

### Identification of cortical tubers

The multifocal tubers were identified by an experienced pediatric neuroradiologist (PEG) and labeled in FreeView (http://surfer.nmr.mgh.harvard.edu/). The tuber closest to the ECDs was then manually segmented in FreeView with margins confirmed by an experienced pediatric neuroradiologist (PEG). The tuber margins were used to determine the distance between the ECDs and the tuber (Section MEG and EEG Data Analysis).

### MEG and EEG data analysis

Both EEG and MEG data were band-pass filtered between 1 and 70 Hz with a notch filter applied at 60 Hz. Bad MEG and EEG channels were excluded from further analysis. Interictal spikes were marked independently in EEG and MEG data by a pediatric neurologist (SC). Marked interictal spikes were revisited in order to identify similar spatiotemporal profiles in the time traces of EEG and MEG. A prominent morphology of epileptiform activity was identified in the data of each modality (see MEG and EEG Results for details). This resulted in two groups of interictal spikes, one for EEG and one for MEG signals. The MEG and EEG signals of the interictal spikes in each group were averaged including ±100 ms around peak latency. For the averaged interictal spikes, we calculated the signal-to-noise ratio (SNR) on the sensor level as 10 times the logarithm of the ratio of the averaged signal power to the averaged noise power. The averaged signal power is the sum of the squared amplitudes of spike activity divided by the duration of the spike activity and the averaged noise power is the averaged squared amplitude of the signal before the spike started. We considered a signal interval of ±30 ms with respect to the interictal spike peak latency. The noise interval was extracted from −500 to −50 ms with respect to the peak latency. The SNR was calculated for all channels and only the maximum value was reported.

A realistic three compartment boundary element method (BEM) model was constructed based on T1-weighted MRI data, for solving the forward problem. The inner skull boundary, the outer skull boundary, and the skin were automatically segmented and triangulated with 5,120 triangles per surface (Haueisen et al., 1997) using the MNE software (http://martinos.org/mne). For source analysis, we co-registered the EEG and MEG sensor configurations with the BEM model. The MNE software defined a head coordinate system based on left pre-auricular point (LPA), Nasion, and right pre-auricular point (RPA) digitization. The head digitization with Fastrack (Polhemus, Colchester, VT, USA) resulted in a digitization set of points on patient’s skin in the head coordinate system including EEG electrode positions. The MRI images and the BEM model were transformed into this head coordinate system. The MEG sensor configuration was defined in the coordinate configuration of the Polaris system (Northern Digital Inc., Waterloo, ON, Canada) used for head digitization in the MSR. Predefined points marked on the patient’s skin served as common points in Polhemus and Polaris digitization. Based on these common points, the MEG sensor configuration was transformed onto the BEM model in the head coordinate system.

Since we do not know *a priori* how extended are the epileptiform generators, we analyzed the MEG and EEG data by using two source localization methods: one that assumes a focal underlying generator that explains the observed MEG/EEG signal (i.e., ECD) and one that presumes extended sources [i.e., minimum norm estimates (MNE) (Hämäläinen and Ilmoniemi, 1994)]. MNE software was used for analyzing the MEG and EEG data. The source localization findings of the two methods were finally compared to each other. Our goal was to quantify the distance between the epileptiform source activity and the margin of a distinct calcified tuber. The whole brain volume provided valid source space for the ECD localization and the MNE reconstruction based on the white–gray-matter boundary. The cortical tuber was a valid source space for the ECD analysis, but not for the MNE analysis. Within the tuber volume, it was not feasible to segment a cortical surface. Therefore, the white–gray-matter boundary passed the calcified tuber volume. ECDs were estimated for each interictal spike at the peak latency of each spike, and for the averaged interictal spike from −15 to 0 ms from the peak latency of the averaged spike in increments of 5 ms. This time-interval represented the upslope of the spike from approximately 50% of the peak amplitude to the peak amplitude. For each ECD localized to the averaged interictal spike, we computed the confidence volume using Curry 7 (Compumedics Neuroscan, Charlotte, NC, USA). For the two dipole-clusters formed by ECDs at the individual interictal EEG and MEG spikes, we performed a principal component analysis (PCA) to estimate a representative ellipsoid for the cluster (Ziolkowski et al., 2002). The eigenvalues of the covariance matrix provided the semi axis of the ellipsoid. The ellipsoid was located in the center of the dipole cluster.

MNE solutions at peak time of the averaged interictal spike were also computed with restriction to the boundary between gray and white matter using the MNE software with default parameters, weighted with dynamic statistical parametric mapping (dSPM) (Dale et al., 2000) and thresholded for display. We calculated the distance from each ECD localization to its closest point on the tuber margin. In order to test whether or not the distance between the cluster of dipoles, fitted to each interictal spike, and the tuber margin differs significantly for MEG and EEG, we performed a Wilcoxon rank sum test with a level of significance of 5% for the dipole tuber distance of the two clusters. Further, we computed the distance between each ECD and the maximum point of the MNE solution.

### DTI data analysis

Diffusion data were processed with Diffusion Toolkit (http://trackvis.org/dtk/) using HARDI/Q-Ball imaging model and second order Runge Kutta propagation algorithm with an angle threshold of 45° and no FA threshold. A three-dimensional segmentation of the tuber file was transformed to create a mirror image of the tuber and then the transformed tuber was manually edited to match the gyral topography of the left hemisphere using FreeView but preserving the same ROI volume. The dipoles cluster formed by the interictal spikes ECDs was used to define the third ROI (each ECD was considered as a cube with 3 voxels edge). ECDs cloud ROI was also transformed to create a mirror image of itself to be used as another ROI of the same volume on the left hemisphere.

The original and transformed tuber volume files, the ECDs cloud, and the transformed ECDs cloud volume files, as well as the T1 and T2 FLAIR images were co-registered with the b0 image using 3D Slicer software (http://www.slicer.org). The tuber, transformed tuber, ECDs cloud, and the transformed ECDs cloud volumes were imported in TrackVis software (http://trackvis.org) as ROI files. Fiber tractography was performed with TrackVis software to create fiber tracks that pass through the tuber, the transformed tuber (i.e., contralateral NAWM), the ECDs cloud (i.e., subcortical white matter adjacent to the tuber within epileptogenic zone), and the contralateral ECDs cloud ROIs. We avoid creating a fourth ROI using the MNE results, since the MNE analysis was performed on the boundary between the gray and white matters. Mean scalar measures of FA, ADC, axial diffusivity (AD), and radial diffusivity (RD) were derived for each fiber track.

## Results

### MEG and EEG results

**Figure 2:**
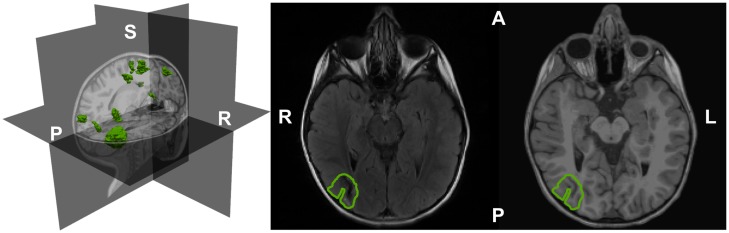
**The segmented and rendered tubers (in green) of the epilepsy TSC patient shown in a 3D head representation based on T1-weighted MRI data (left)**. Tuber outline axial on T2 (center) and T1-weighted MRI images (right). A: anterior; L: left; P: posterior; R: right; S: superior.

Figure 2 shows all the identified tubers in the patient’s brain overlaid on the T1 and T2-weighted structural MRI data. The largest tuber was identified in the right parietal-occipital area with mineralization. Very frequent right posterior temporal/occipital sharp waves (mainly at electrodes P8, P4, and CP6) were observed in EEG signal. These sharp waves always presented the same spatiotemporal profile at the sensor level. Sharp waves and polyspikes were observed from left frontal regions in EEG, but their spatiotemporal map did not consistently present the same topography. For this reason and also due to the presence of predominant epileptic activity in the right posterior quadrant including the occurrence of epileptogenic seizures, we focused our MEG and EEG analysis of simultaneously recorded data on the interictal activity at the right posterior temporal/occipital region.

**Figure 3:**
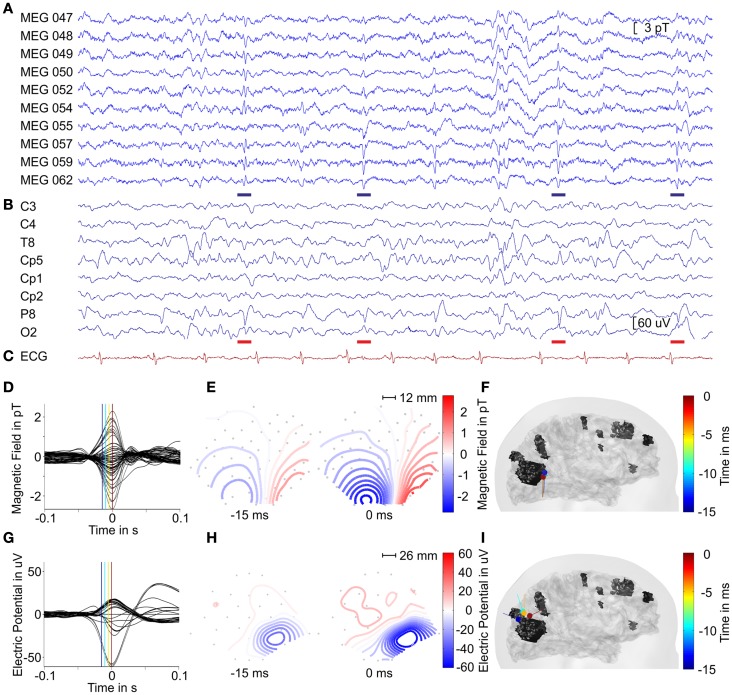
**MEG (A) and EEG (B) signals of selected channels and the ECG (C) trace with interictal spikes marked by blue and red bars indicating epileptiform in MEG and EEG in a time-interval of 10 s**. Averaged interictal MEG **(D)** and EEG **(G)** spikes. The magnetic field maps **(E)** and electric potential maps **(H)** depicted at labeled time points [blue and red lines in **(D,G)**] of averaged signals. The gray dots indicate sensor positions with a geometrical scale as labeled. For positioning with respect to the head, please compare to Figure 1. **(F,I)** Lateral views on the head model with ECD traces of averaged interictal spikes with color coded time information from −15 to 0 ms with respect to peak latency for MEG and EEG, respectively.

Within our region of interest in the right parietal-occipital lobe, we identified a total of 304 interictal spikes of which 135 (44% of total spikes) were detected on EEG and MEG within a time frame of ±15 ms between the peak times. These were considered simultaneous spikes. Eighty-five spikes (28% of total spikes) were identified only on MEG, and 84 (28% of total spikes) were uniquely in EEG traces. This resulted in 220 MEG (72% of total) and 219 EEG (72% of total spikes) spikes. An overview of the number of spikes identified in our recordings is given in Table 1. Figure 3 shows 10 s of filtered data from selected MEG and EEG channels. The colored markers indicate the interictal spikes in each modality providing high signal to noise ratio (SNR) at sensor level.

**Table 1:** **Number of spikes identified in the data of MEG and EEG channels covering the right parieto-occipital lobe**.

	Number	Percentage
Total	304	100
MEG	220	72
EEG	219	72
Common	135	44
Unique MEG	85	19.4
Unique EEG	84	19.1

Two groups of interictal spikes were identified independently among the total MEG spikes and the total EEG spikes according to their localization and morphology in the time traces. MEG spikes (blue bars in Figure 3) were selected when they presented a polarity reversal between MEG channels 054 and 057. They had a mean duration of ~55 ms. EEG spikes were selected if they had a polarity reversal between EEG channels P8 and O2, and were part of a spike wave complex. Spike duration was ~70 ms; the spike wave complex had a mean duration of ~250 ms. This selection of spikes resulted in 46 spikes in EEG and 57 spikes in MEG, with 17 of them seen in both modalities. Within the group of interictal MEG spikes, we found the SNR on a level of 11.1 ± 2.5 dB and the averaged MEG spike reached a SNR of 21.6 dB. The group of interictal EEG spikes provided a SNR of 11.7 ± 3.1 dB and the averaged EEG spike reached a SNR of 25.6 dB.

**Figure 4:**
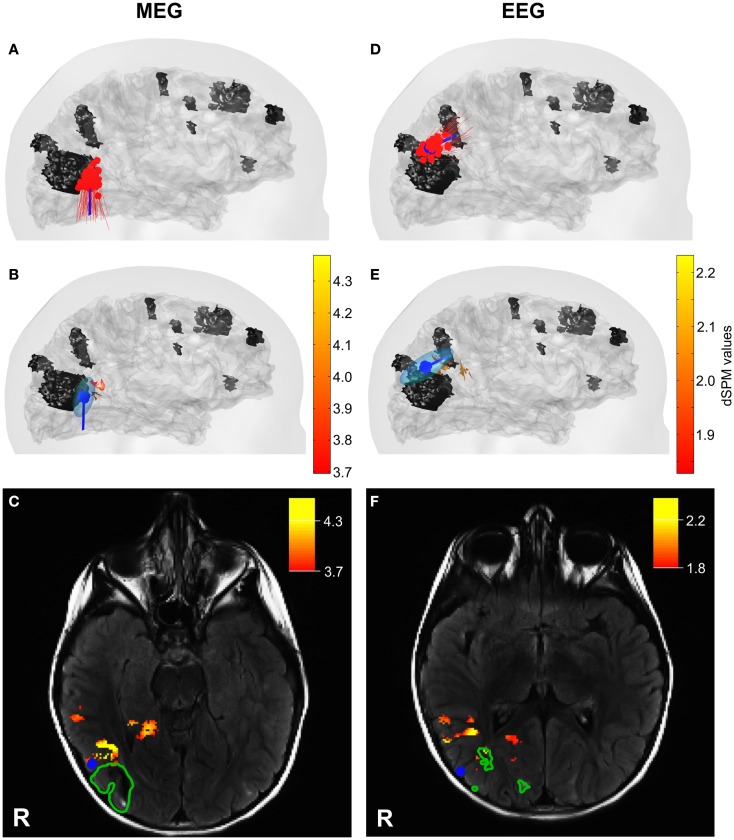
**ECD and MNE localizations for interictal MEG (left column) and EEG (right column) spikes**. **(A,D)** Lateral views on the head model with ECD localization for each interictal spike (red) and the averaged interictal spikes (blue). **(B,E)** Lateral views on the head model with MNE localizations (red – yellow scale) and ECD for averaged interictal spikes (blue) with ellipsoid representing the dipole-clusters. The tuber volumes are indicated in black. Skin and cortical surface are indicated in gray. **(C,F)** Axial slices of MRI FLAIR images with tuber margins (green), ECD localizations for averaged interictal spike (blue dot), and MNE localizations (red – yellow scale). R: Right.

Since our MEG system provides partial head coverage, our initial effort was to ensure that the sensor array covered both the minima and maxima of the epileptiform activity. At sensor level, we found lateralized minima on the edge of the sensor array for both MEG and EEG configurations (see Figure 3) with respect to the tuber localization, which is in agreement with Ochi et al. (2011). Figure 4 shows the ECD and MNE localizations for the averaged interictal spikes. Both ECDs and MNE maxima were localized in the immediate vicinity of the calcified tuber.

The cluster formed by the ECDs fitted to the 57 interictal MEG spikes was localized on average in a distance of 4 ± 2 mm laterally with respect to the tuber margin (red dipoles in Figure 4A). The equivalent ellipsoid of the dipole cluster circumscribed a volume of 1.9 cm^3^ (Figure 4B). The analysis of the averaged MEG spike from −15 to 0 ms with respect to peak latency provided a stable magnetic field with respect to its orientation and increased in intensity during the considered time-interval (Figure 3E). The dipoles localized to the averaged MEG spike generated a 4.5 mm trace in inferior direction anterior to the calcified tuber (Figure 3F). The distance between the ECD localizations and the tuber margin decreased from ~5 to ~3 mm (3.8 ± 0.8 mm). The dipole localized to −15 ms provided a confidence volume of 2.9 cm^3^ and the subsequent dipoles provided confidence volumes of 0.4 ± 0.15 cm^3^. The ECD fitted to the peak of the averaged interictal MEG spikes was localized ~3 mm anterior lateral to the calcified tuber margin and provided a confidence volume of 0.4 cm^3^ (blue dipole on Figures 4B,C). The ECDs fitted to the averaged interictal MEG spikes moved across time from −15 to 0 ms staying always at the periphery of the tuber’s volume with a superior–inferior direction by the lateral side of the calcified tuber (Figure 3F). The dipole fitted to peak latency reached the maximal goodness of fit (GOF) of 90% with an amplitude of 72.9 nAm. The maximum point of the MNE distribution localized ~4 mm anterior to the calcified tuber margin (Figures 4B,C). The distance between the center of the ECD cluster and the ECD localization for the averaged interictal MEG spikes was less than 0.5 mm. The distance between the ECD localization of the averaged interictal MEG spike and the maximum point of the MNE localization was ~11 mm. The difference between the ECD orientation for the averaged interictal MEG spike and the surface normal for the maximum point of the MNE localization was 17.2°. The distance between the ECD cluster center and the MNE localization was ~11 mm, as well.

The ECDs fitted to each of the 46 interictal EEG spikes were localized on average at a distance of 5 ± 2 mm superior to the tuber margin (red dipoles in Figure 4D). The equivalent ellipsoid of the dipole cluster circumscribed a volume of 3.8 cm^3^ (Figure 4E). The analysis of the averaged EEG spike provided a negative pole over the right parietal-occipital region as main feature, which increased in extend and slightly changed its morphology during upslope of the spike from −15 to 0 ms with respect to peak latency (Figure 3H). The dipole trace localized to the averaged EEG spike started superior posterior to the calcified tuber in a distance of ~12 mm to the tuber margin with a dipole orientation in posterior direction. The subsequent dipoles moved ~12 mm anterior and changed orientation to a mainly anterior direction (Figure 3I). The distance between the dipole localizations and the tuber margin remained with ~14 and ~12 mm relatively stable (−15 to −5 ms). The dipoles localized to the averaged EEG spikes provided confidence volumes of 11 ± 6 cm^3^. The ECD fitted to the averaged interictal EEG spikes was localized at a distance of ~5 mm posterior superior to the calcified tuber margin and provided a confidence volume of 12.6 cm^3^ (blue dipole in Figures 4E,F). The ECDs fitted to the averaged interictal EEG spikes moved slightly across time (from −15 to 0 ms) staying always at the periphery of the tuber’s volume in an anterior–posterior direction and turned ~90°(Figure 3H). The ECD trace stabilized between 0 and +10 ms. The dipole fitted to peak latency reached the maximal GOF of 70% with an amplitude of 93.6 nAm. The maximum point of the MNE distribution localized ~9 mm away from the margin outside the volume of the calcified tuber (Figures 4E,F). The distance between the center of the ECD cluster and the ECD localization for the averaged interictal EEG spikes was ~2 mm. The distance between the ECD localization of the averaged interictal EEG spike and the maximum point of the MNE localization was ~14 mm. The difference between the ECD orientation for the averaged interictal MEG spike and the surface normal for the maximum point of the MNE localization was 38.7°. The distance between the ECD cluster center and the maximum point of the MNE localization was ~12 mm.

The dipole cluster localized by MEG was significantly closer (*p* < 0.05) to the tuber’s margins compared to the one localized by EEG. The distance between the centers of the two ECD clusters for interictal MEG and EEG spikes was ~22 mm. Similarly, the ECDs fitted to the averaged interictal MEG and EEG spikes localized ~22 mm apart. The maximum points of the MNE localizations for the averaged interictal MEG and EEG spikes localized in a distance of ~24 mm to each other.

### DTI results

**Figure 5:**
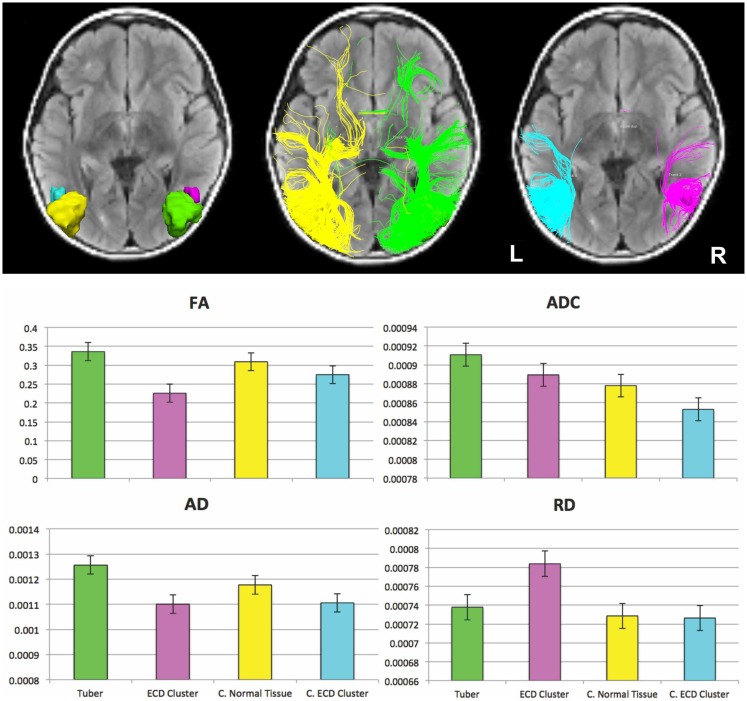
**(Upper panel)** Fiber tracts passing through the four ROIs. (Left-upper panel) ROIs displayed on an axial FLAIR image: ROI encompassing the cortical and subcortical component of the large right-sided calcified tuber (green), ROI of same volume encompassing normal appearing tissue contralateral to the tuber (yellow) modified to respect gyral folds, ROI encompassing the ECD cluster formed by the MEG dipoles resulting from fitting the interictal spikes (magenta), and ROI of same volume encompassing the normal appearing tissue contralateral to the ECD cluster (cyan). (Middle-upper panel) Fibers passing through the ROIs defined by the tuber (green) and the normal appearing tissue contralateral to the tuber (yellow). (Right-upper panel) Fibers passing through the ECD cluster (magenta) and the normal appearing tissue contralateral to the ECD cluster (cyan). **(Lower panel)** Mean (±SE) for the scalar values of mean FA, ADC, AD, and RD for fiber tracks passing through the large cortical tuber (green), the ECD cluster formed by the dipoles fitted at interictal spikes (magenta), the normal appearing tissue contralateral to the tuber (yellow), and at the normal appearing tissue contralateral to the ECD cluster (cyan).

Figure 5 (upper panel) presents the four ROIs and the corresponding fibers passing through them. Figure 5 (lower panel) presents the mean for the scalar values of mean FA, ADC, AD, and RD for fiber tracks passing through the four ROIs. The tracks passing through the ECD cluster had the lowest mean FA and the highest mean RD values. The tracks passing through the tuber showed the highest mean FA and highest mean AD values. The mean FA, ADC, and AD values were higher for the tuber tracks than for the ECD cluster tracks with tracks on the contralateral side showing the same pattern. However, the difference between the mean FA, ADC, and AD values for the tuber tracks and the ECD cluster tracks was more pronounced than the difference between the contralateral ROI tracks. Moreover, the increase in the mean RD value observed for the ECD cluster tracks compared with the tuber tracks on the right hemisphere was not observed between the tracks of the left hemisphere. Finally, mean ADC values were higher all together for the tuber tracks and the ECD cluster tracks than the mean ADC values of the contralateral tracks.

## Discussion

In this illustrative case, we localized predominant epileptiform brain activity in a 4-year-old child with epilepsy due to TSC by using pediatric MEG, EEG, and DTI. The predominant epileptiform activity, when analyzed by simultaneously recorded MEG and EEG, was consistently localized to the tissue surrounding the tuber rather the tuber itself. DTI results further support the notion that epileptogenicity may come from the surrounding tissue of the tuber, since, they indicate different microstructural features for the white matter tracks passing through the region of electrophysiologic abnormality localized in the vicinity of the tuber compared to the other ROIs. Additional studies are needed to determine if this is a consistent finding in large numbers of TSC patients.

Previous clinical epilepsy studies indicate that epileptiform activity is sometimes visible only in MEG or in EEG (Baumgartner et al., 2000; Yoshinaga et al., 2002; Rodin et al., 2004; Iwasaki et al., 2005). The two techniques provide different sensitivity profiles depending on the orientation and depth of the underlying source (Goldenholz et al., 2009; Ahlfors et al., 2010; Haueisen et al., 2012). For the achievement of a complete picture of the underlying epileptogenic sources, it has been recommended that these two modalities are used simultaneously in epilepsy studies (Nakasato et al., 1994; Stefan et al., 2003). Here, we used both high-resolution MEG and EEG for two reasons: (i) to capture epileptiform activity visible only from a single modality, and (ii) to obtain a complete picture of the entire brain epileptiform activity, since our MEG system has a partial coverage of the head.

In our study, the fraction of the commonly detected interictal spikes in both EEG and MEG was comparable to previous studies [i.e., here 44 vs. 41% in Ramantani et al. (2006) and 51.1% in Park et al. (2004)]. For source localizations to interictal epileptiform activity, it is recommended to perform the analysis during the upslope of the spike since source propagation can already occur during this interval (Alarcon et al., 1994; Lantz et al., 2003; Ray et al., 2007). The ECDs localizations were performed for the averaged interictal MEG and EEG spikes from −15 to 0 ms with respect to the peak latency in intervals of 5 ms. This time-interval represented the upslope of the spike from approximately 50% to the maximum amplitude of the interictal spike. For MEG, the field remained stable with respect to its orientation and increased in intensity during the considered time-interval. The MEG dipole trace remained relatively stable across time during the upslope of the averaged signal. For EEG, the dipole trace presented a slight propagation in anterior direction with an orientation change from posterior to anterior, but the dipoles were always localized outside the calcified tuber in millimeter distances. The dipoles at the peak of the averaged EEG and MEG spikes provided the smallest distance to the tuber margin, and therefore provided the critical configuration for testing our hypothesis. Based on these observations, we performed the localization of single-spikes at the peak time in order to achieve ECDs with relatively high GOF considering the high SNR at this time point.

The ECD localizations of interictal MEG and EEG spikes formed ECD clusters similar to those reported by Iida et al. (2005), Kamimura et al. (2006), Sugiyama et al. (2009), and Widjaja et al. (2010). The generators identified by MEG located closer to the tuber’s margins compared to those localized by EEG, which is in line with previous source localization findings in TSC-related epilepsy patients (Jansen et al., 2006; Xiao et al., 2006). ECDs and MNE localization results were in agreement; both were localized to the vicinity of the single calcified tuber (see Figure 4). Differences in the localization results of the two source localization methods can be due to the fact that the MNE solutions were constrained to the cortical surface, while ECDs solutions were unconstrained. This hypothesis was supported by the small deviations between the ECD orientations for the averaged interictal spikes and the surface normal for the maximum point of the MNE localizations. The different localization results for MEG and EEG with respect to the calcified tuber may indicate different epileptogenic foci associated to the tuber. These two neuroimaging modalities prefer different source orientation, which can result in the identification and localization of different source aspects. In the present case, the dipole localizations present tangential orientation for interictal MEG spikes and mainly radial orientation for interictal EEG spikes. A direct comparison between the MEG and EEG source localization results cannot be performed, since different number of sensors were used from the two neuroimaging modalities. Indeed, an explanation for the superior localization of the sources for EEG data compared to sources for MEG data could be provided by the fact that our electrode configuration contained very few electrodes below the hairline. In temporal lobe epilepsy, Sperli et al. (2006) indicated a shift of source localizations to dorsal structures when inferior temporal electrodes were not included in the recording setup. Further, the minima of the electric potential and magnetic field maps located on the edge of the sensor arrays and the maxima were incompletely circumscribed. Such an incomplete sampling of the electric potential and magnetic field could potentially lead to erroneous source localizations (Michel et al., 2004).

However, our findings are in agreement with previous studies indicating that epileptogenic tissue may be predominately localized in the surroundings of cortical tubers (Weiner, 2004; Xiao et al., 2006; Major et al., 2009). The high sensitivity and excellent localization accuracy of our MEG system allowed us to detect and localize accurately the epileptiform activity with respect to the location of the calcified tuber. The localization of single-spikes and averaged spikes were consistent; the two clusters for MEG and EEG were localized in a relatively small region outside tuber’s margins. ECD traces across time indicated propagating epileptiform foci localized in the vicinity of a calcified tuber but never passed the tuber’s margins going inside the tuber.

The view that epileptiform activity is generated in the surrounding tissue of tubers is supported by both clinical as well as animal studies, which indicate that cortical tubers may not be essential for epileptogenicity. A significant improvement in the seizure profiles of TSC patients has been observed after the resection of these single epileptogenic tubers and their surrounding tissue (Guerreiro et al., 1998; Koh et al., 2000; Lachhwani et al., 2005). Kaufmann et al. (2009) reported a tuberless TSC infant with intractable epilepsy, while cortical synaptic hyperexcitability in the absence of cortical tubers was reported by Wang et al. (2007) in animals.

Our DTI results support the notion that the white matter tracts associated with the region of electrophysiologic abnormality (ECD cluster) have microstructural features that may be distinct from tracts associated with the tuber and tracts generated from a similar ROI in contralateral normal appearing tissue. Decreased FA due to increased RD noted in the ECD cluster may be a sign of disorganized, demyelinated, dysmyelinated, and/or poorly myelinated axons (Beaulieu and Allen, 1994; Gulani et al., 2001; Song et al., 2002, Nair et al., 2005; Song et al., 2005). Similar findings to our ECD cluster were reported in the NAWM surrounding the cortical tubers by Widjaja et al. (2010) who suggested that these abnormal diffusivity values may reflect cortical dysplasia or could be related to ictal and/or interictal activity. These results are also in accordance with animal studies where recurrent seizures are shown to cause impairment in myelin development (Dwyser and Wasterlain, 1982; Song et al., 2003).

The potential etiologies of increased ADC and increased FA in the tuber primarily due to increased AD are less clear. Although prior studies have suggested that reduced axonal density or caliber could increase the extra-axonal space allowing faster water molecule movement parallel to axons (Kumar et al., 2008, 2010; Sun et al., 2008), this explanation is unsatisfying in our case as RD is not increased. Increased ADC values in epileptogenic tubers have been previously reported by Jansen et al. (2003). Here, we provide additional information for tracks passing through the tuber ROI. Prior studies have suggested that increased ADC might be reflective of hypomyelination due to loss of barriers to water motion (Chandra et al., 2006), again the lack of an increase in RD makes this interpretation unsatisfactory.

The present study is an illustrative case of the combined application of MEG and DTI in a single TSC pediatric patient. Our findings support the view that epileptogenicity in TSC patients may be derived from abnormally developed cortex surrounding tubers, but are not conclusive. For MEG, we used an innovative system especially designed for pediatric use that offers better localization accuracy and sensitivity compared to the adult conventional systems for a specific ROI. We provide a detailed and extensive analysis of our data making use of four different neuroimaging modalities (i.e., MEG, EEG, MRI, and DTI). Data from neurophysiological methods, such as MEG and EEG, were combined with data from DTI and MRI to examine how the epileptiform activity is coupled with anatomical changes. Although none of the observations made in this case study is strong enough to completely rule out alternative interpretations, the converging evidence of the independently used neuroimaging modalities makes our hypothesis the most parsimonious explanation and sets the stage for a larger study.

## Conflict of Interest Statement

The authors declare that the research was conducted in the absence of any commercial or financial relationships that could be construed as a potential conflict of interest.
